# Supplementary material for: From STRs to SNPs via ddRAD‐seq: Geographic assignment of confiscated tortoises at reduced costs
Source: Evol Appl. 2022 Aug 31;15(9):1344–59. doi: 10.1111/eva.13431 (PMC9488678; doi:10.1111/eva.13431)
Supplement: Supplementary file 1 — Figure S1–S12 [file EVA-15-1344-s001.docx]

**FIGURE S1**. Schematic diagram representing the workflow of this study.

**
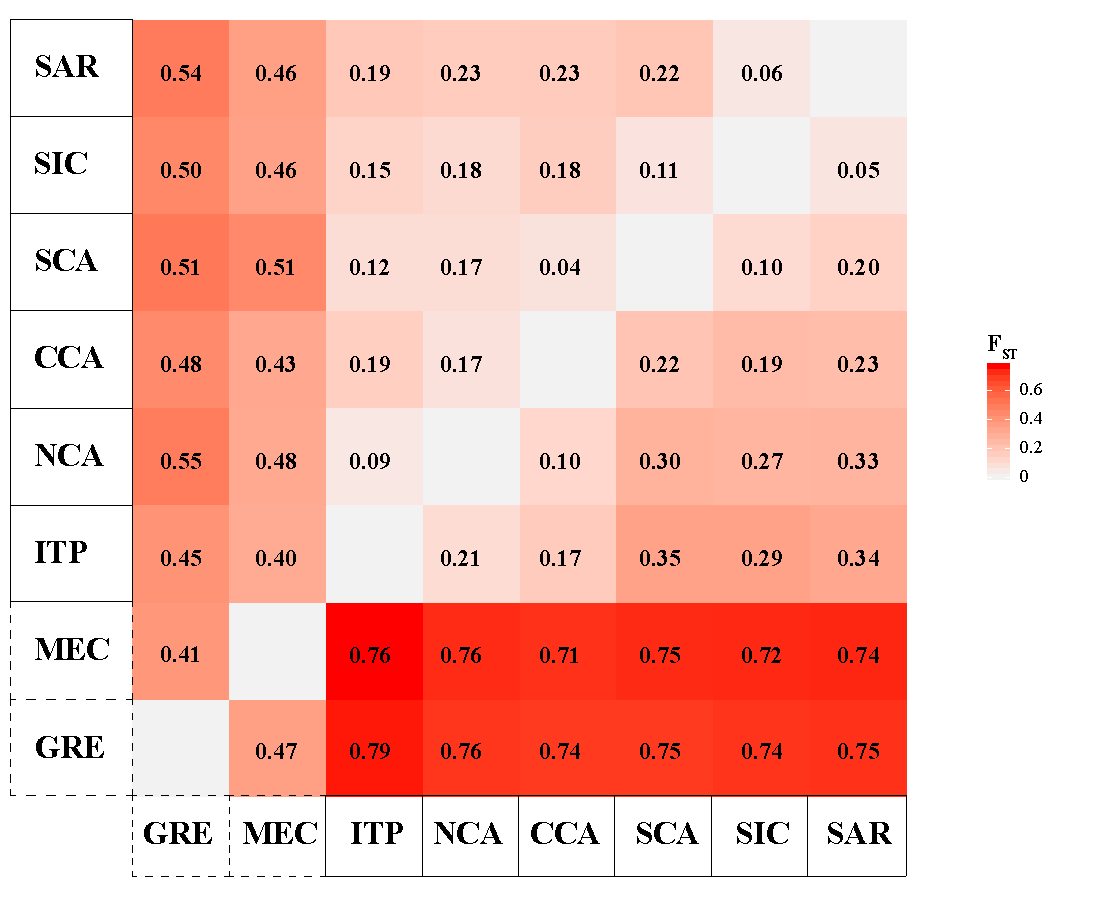
**

**FIGURE S2**. F_ST_ values. SNPs (from ddRAD-seq) (below diagonal) and STRs (above diagonal). GRE=Greece; MEC=Mesola and Croatia; ITP=Italian Peninsula; NCA=Northern Calabria; CCA=Central Calabria; SCA=Southern Calabria; SIC=Sicily; SAR=Sardinia. The two subspecies *Testudo hermanni hermanni*: solid line, *Testudo hermanni boettgeri*: dashed line.


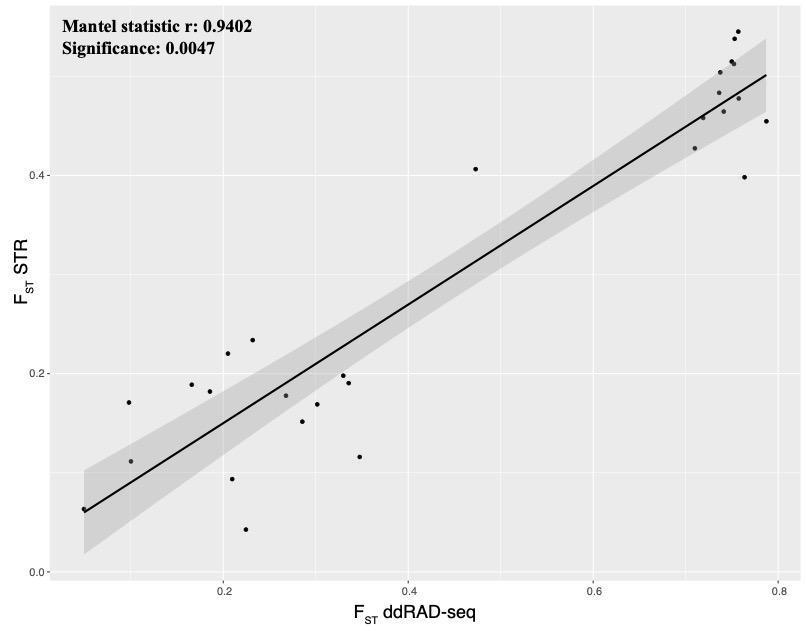


**FIGURE S3**. Mantel test ﻿comparing F_ST_ values between SNPs (ddRAD-seq) and STRs (Biello et al., 2021) datasets.


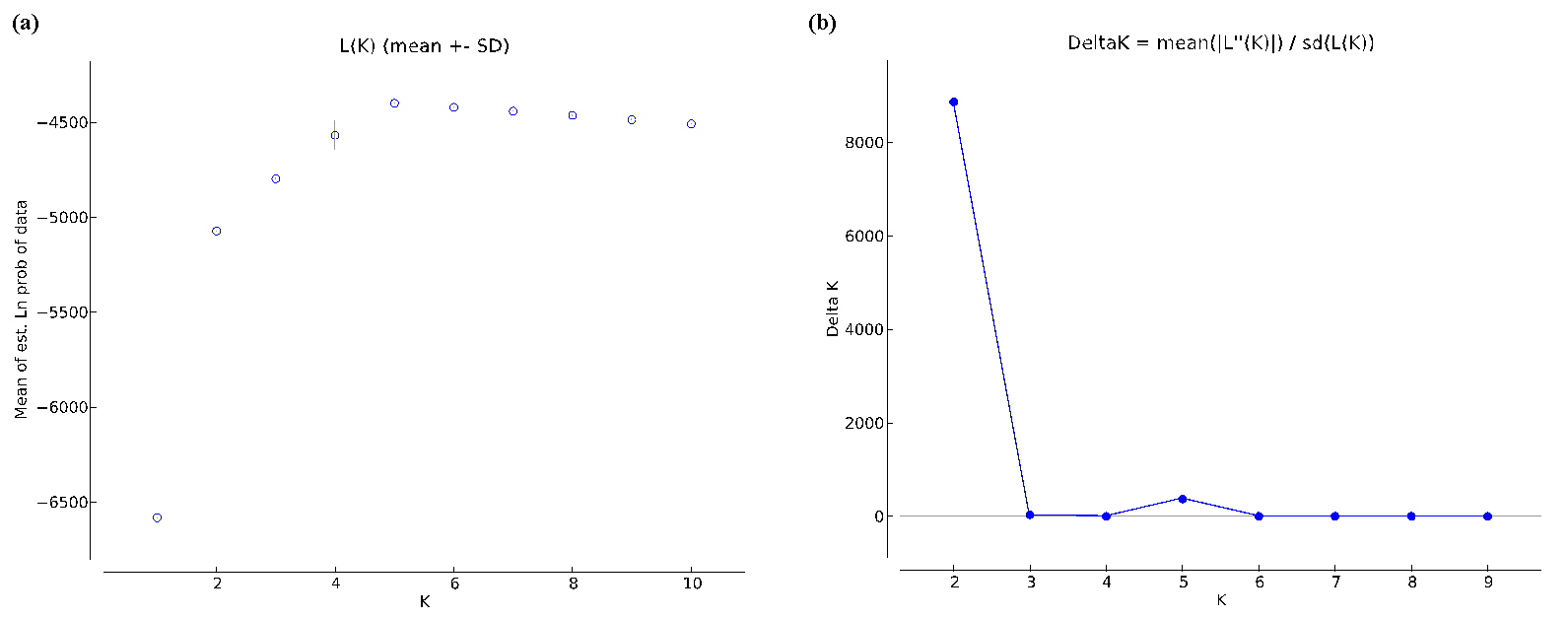


**FIGURE S4**. Probability of number of clusters (K) for 292 samples of *Testudo hermanni*, analysed at 7 STRs loci, using STRUCTURE HARVESTER (Earl et al., 2012). The Ln likelihood value (a) as described by Pritchard et al (2000) and the Delta K method (b) by Evanno et al. (2005).


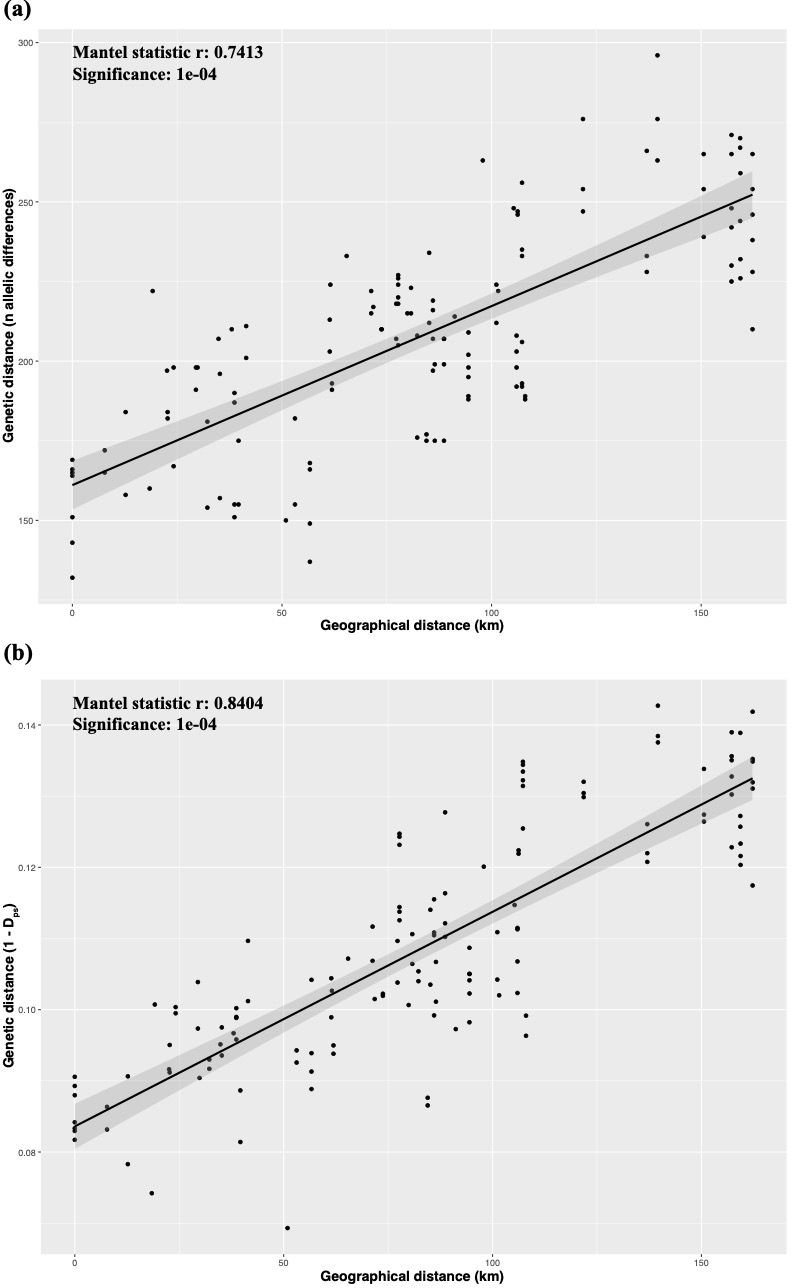


**FIGURE S5**. Mantel test ﻿comparing Euclidean distance (km) and number of allelic differences (a) and D_PS_ (b).


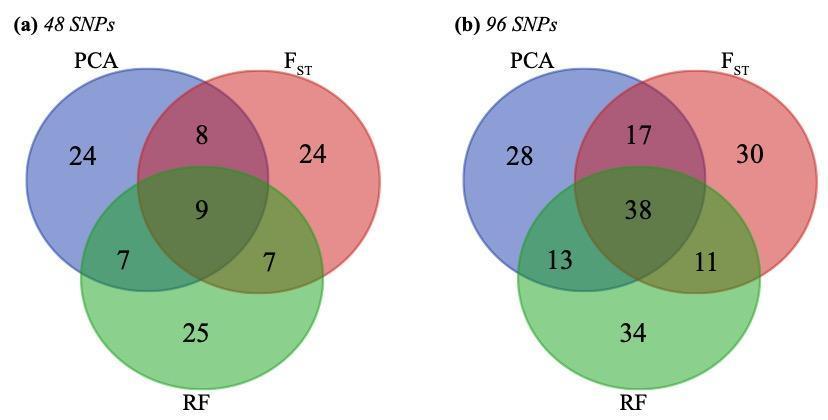


**FIGURE S6**. Venn diagram of SNPs shared among panel classes of 48 SNPs (a) and 96 SNPs (b).


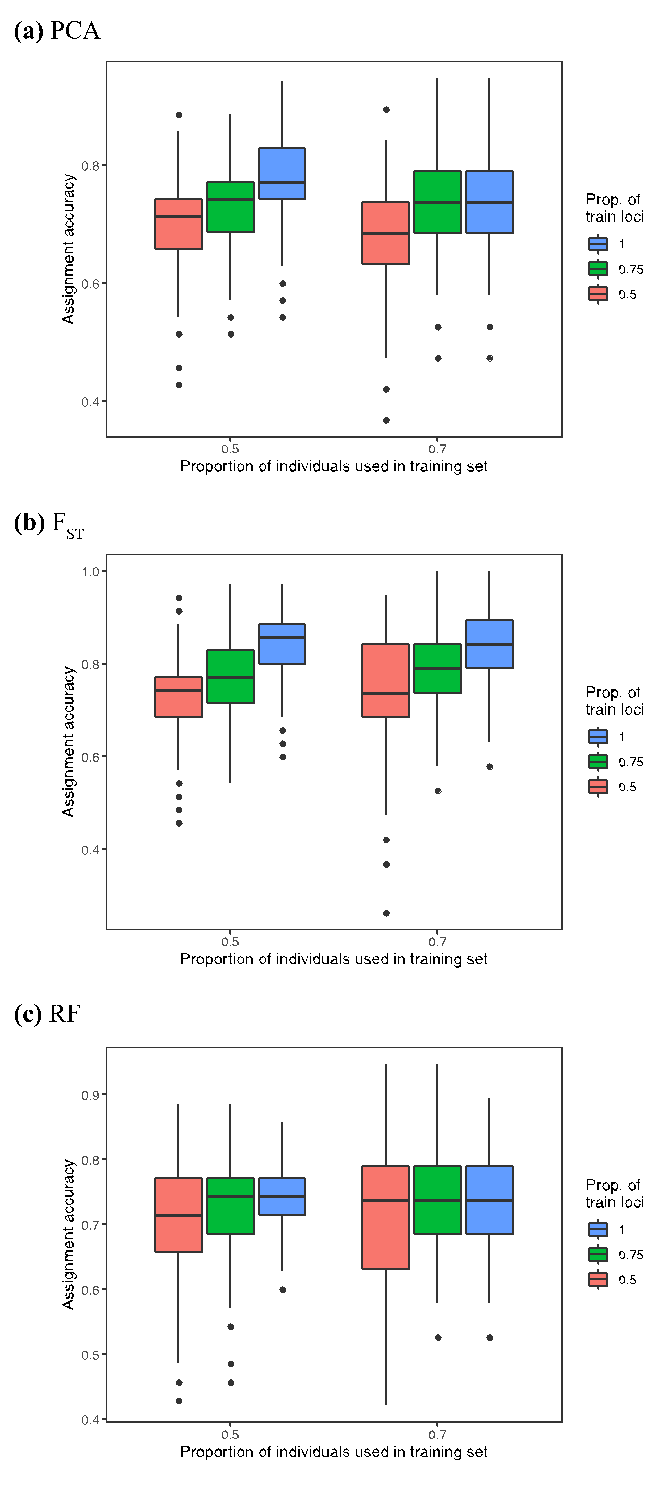


**FIGURE S7.** Overall assignment accuracies to the eight genetic clusters estimated via Monte Carlo cross‐validation of the 48 SNPs panels, with two levels of training (baseline) individuals (50% and 70% of individuals from each group) crossed by up to three levels of training loci (top 50%, 75% and all loci) by 500 resampling events: (a) PCA; (b) F_ST_; and (c) RF.

**
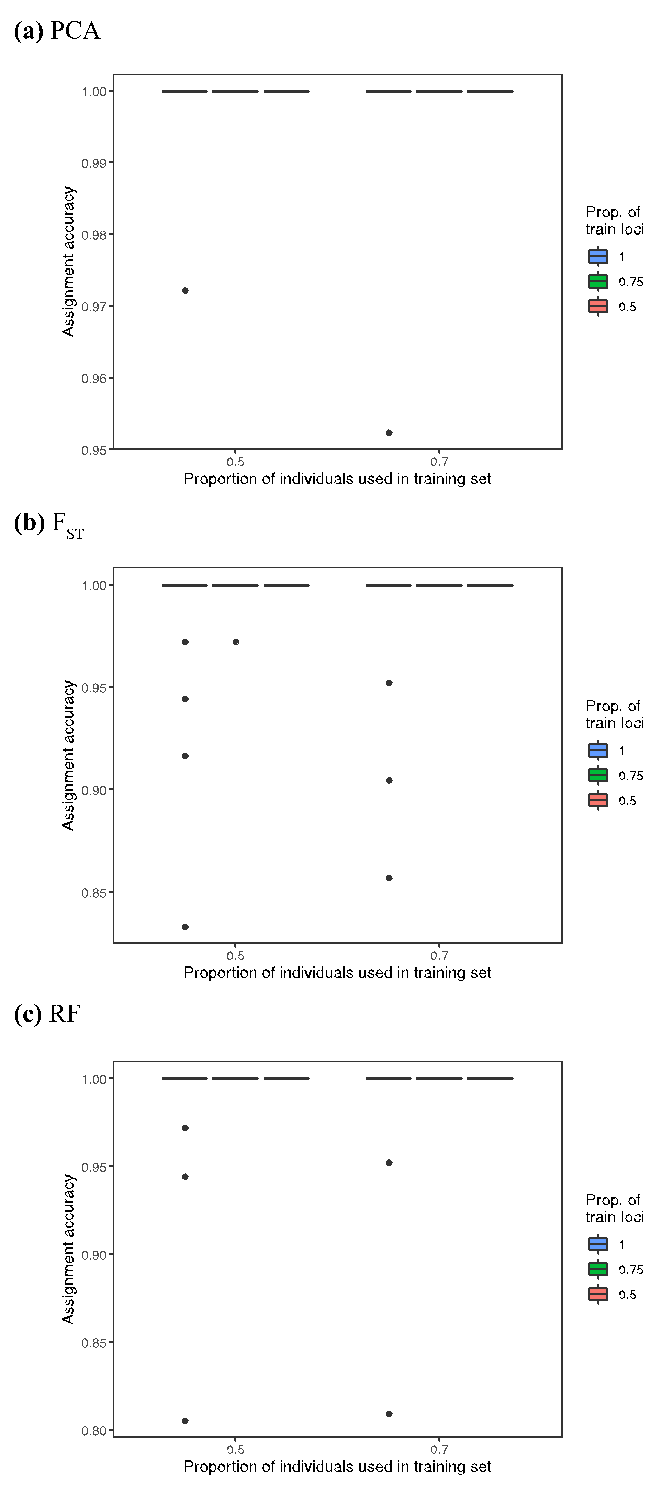
**

**FIGURE S8**. Overall assignment accuracies to the two subspecies (*T. h. boettgeri* and *T. h. hermanni*) estimated via Monte Carlo cross‐validation of the 48 SNPs panels, with two levels of training (baseline) individuals (50% and 70% of individuals from each group) crossed by up to three levels of training loci (top 50%, 75% and all loci) by 500 resampling events: (a) PCA; (b) F_ST_; and (c) RF.

**
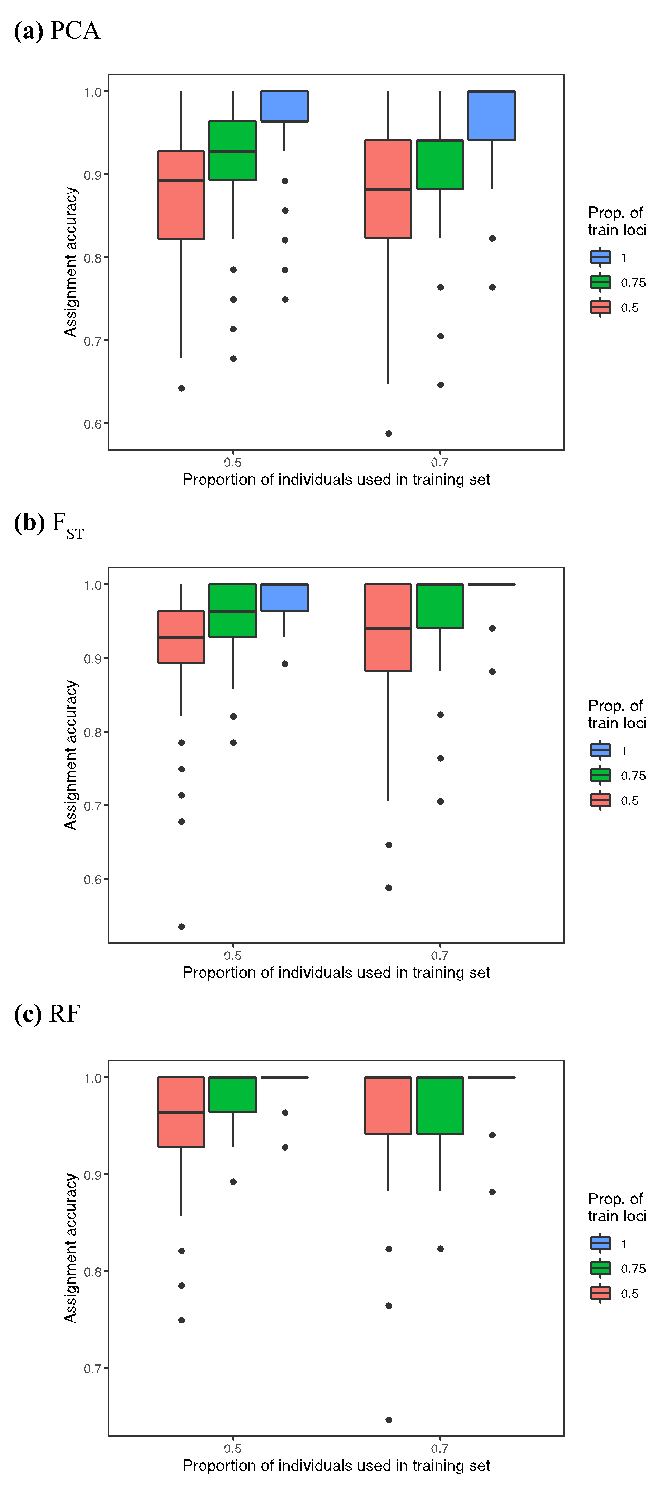
**

**FIGURE S9**. Overall assignment accuracies to the two main *T. h. hermanni* groups, Italian Peninsula (ITP, NCA and CCA) and Mediterranean Islands (SCA, SIC and SAR), estimated via Monte Carlo cross‐validation of the 48 SNPs panels, with two levels of training (baseline) individuals (50% and 70% of individuals from each group) crossed by up to three levels of training loci (top 50%, 75% and all loci) by 500 resampling events: (a) PCA; (b) F_ST_; and (c) RF.


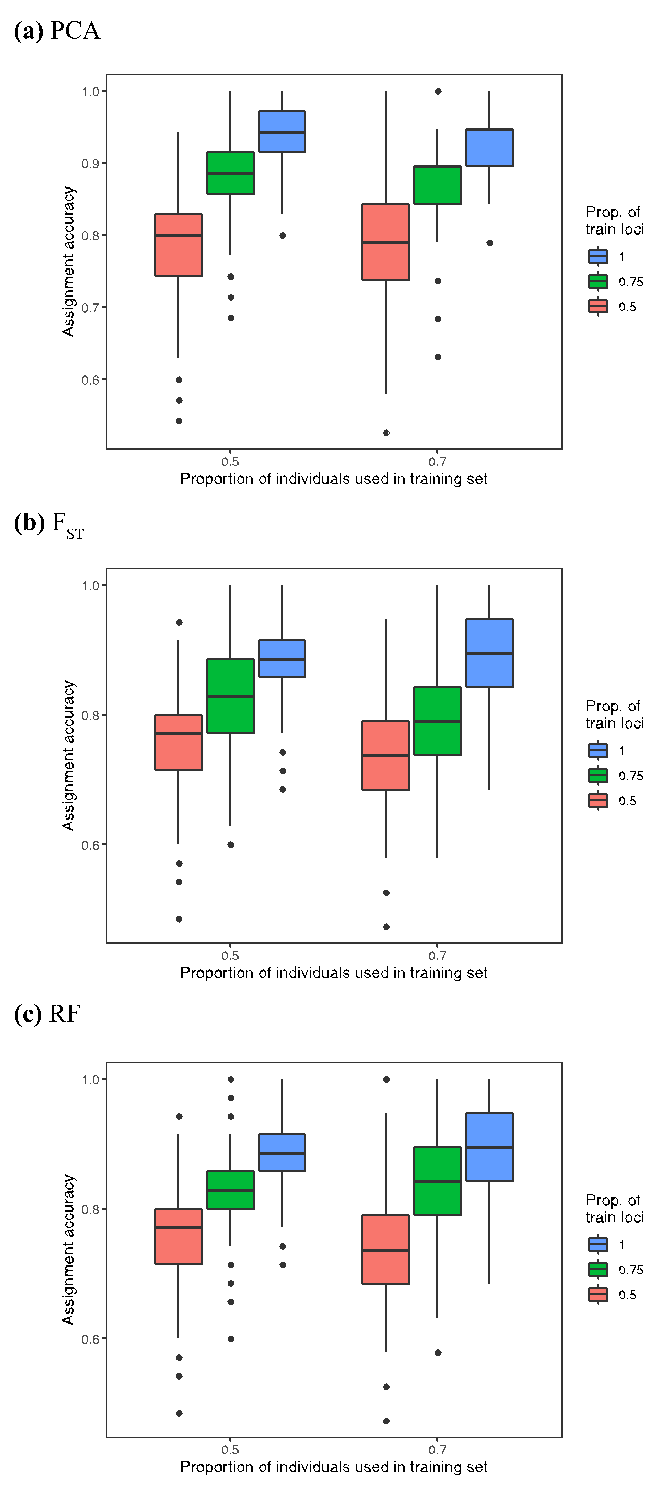


**FIGURE S10**. Overall assignment accuracies to the eight genetic clusters estimated via Monte Carlo cross‐validation of the 96 SNPs panels, with two levels of training (baseline) individuals (50% and 70% of individuals from each group) crossed by up to three levels of training loci (top 50%, 75% and all loci) by 500 resampling events: (a) PCA; (b) F_ST_; and (c) RF.

**
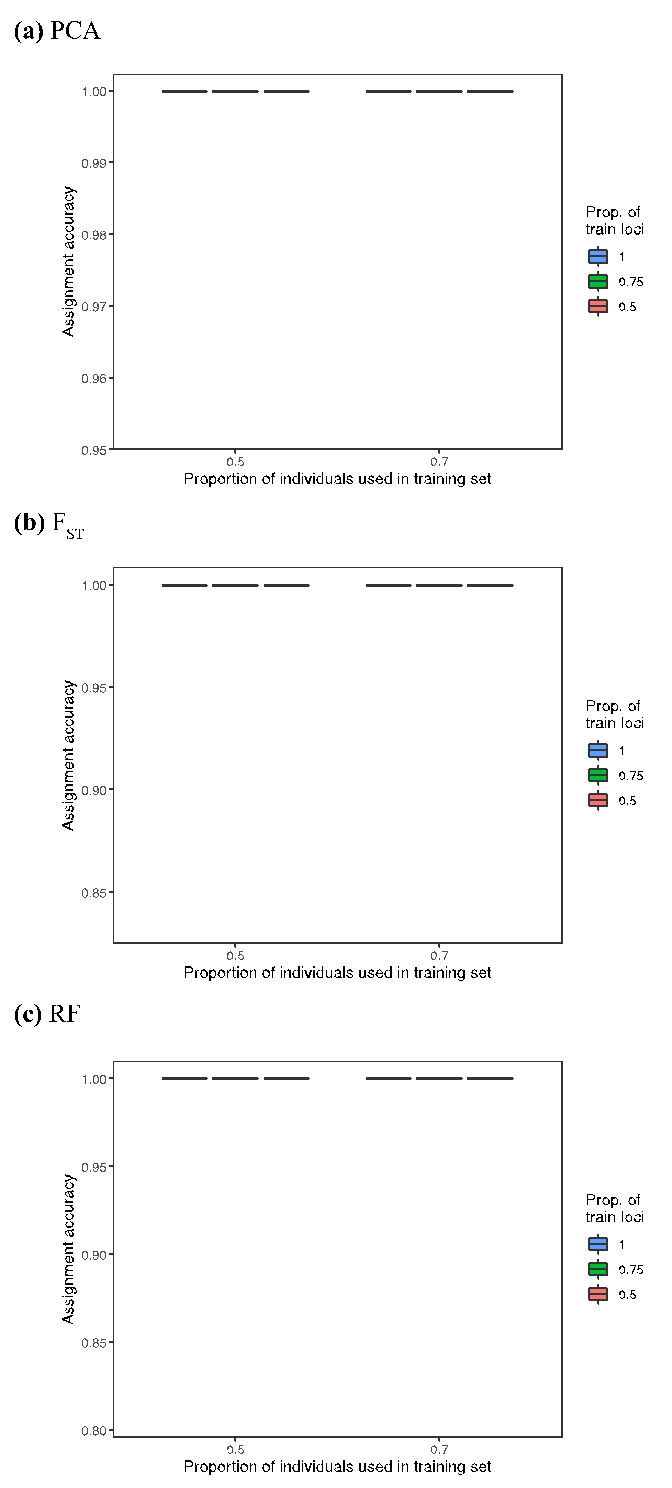
**

**FIGURE S11**. Overall assignment accuracies to the two subspecies (*T. h. boettgeri* and *T. h. hermanni*) estimated via Monte Carlo cross‐validation of the 96 SNPs panels, with two levels of training (baseline) individuals (50% and 70% of individuals from each group) crossed by up to three levels of training loci (top 50%, 75% and all loci) by 500 resampling events: (a) PCA; (b) F_ST_; and (c) RF.

**
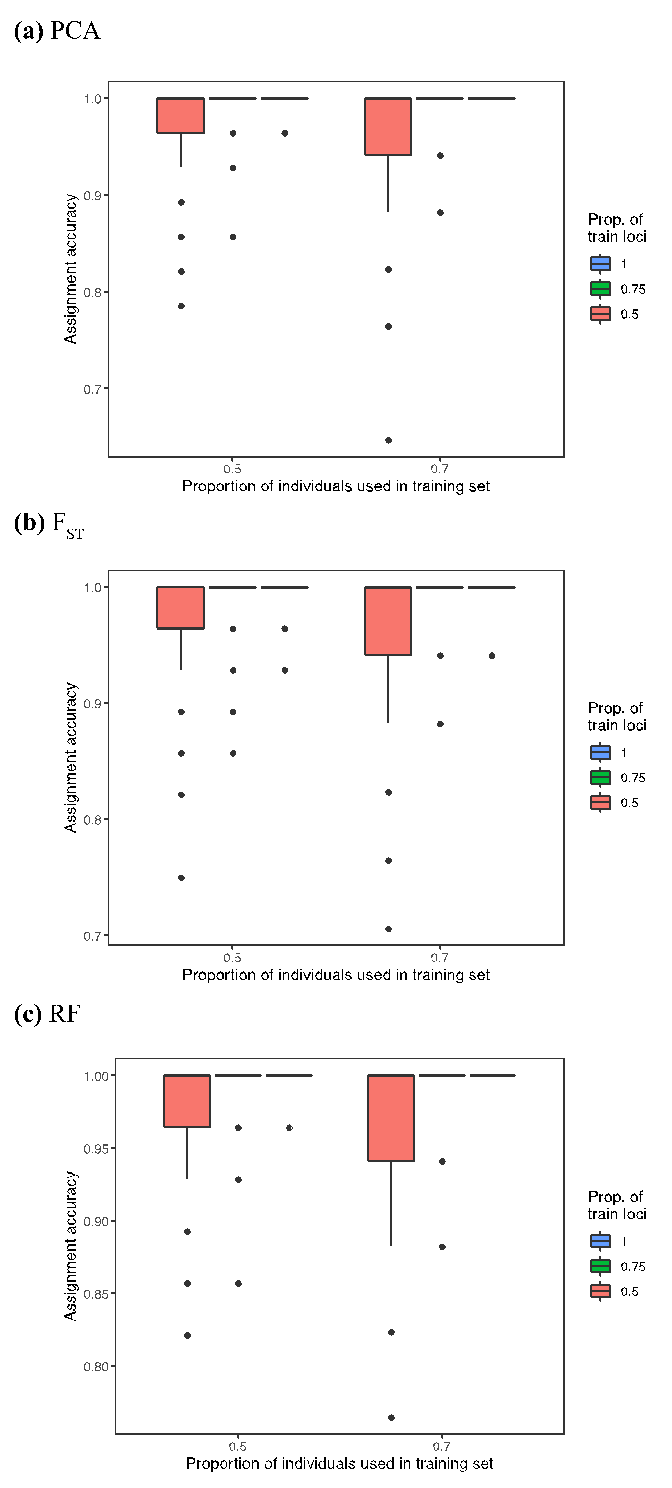
**

**FIGURE S12**. Overall assignment accuracies to the two main *T. h. hermanni* groups, Italian Peninsula (ITP, NCA and CCA) and Mediterranean Islands (SCA, SIC and SAR), estimated via Monte Carlo cross‐validation of the 96 SNPs panels, with two levels of training (baseline) individuals (50% and 70% of individuals from each group) crossed by up to three levels of training loci (top 50%, 75% and all loci) by 500 resampling events: (a) PCA; (b) F_ST_; and (c) RF.

**REFERENCES**

Biello, R., Zampiglia, M., Corti, C., Deli, G., Biaggini, M., Crestanello, B., … Canestrelli, D. (2021). Mapping the geographic origin of captive and confiscated Hermann’s tortoises: A genetic toolkit for conservation and forensic analyses. *Forensic Science International: Genetics*, *51*, 102447. https://doi.org/10.1016/j.fsigen.2020.102447

Earl, D. A., & vonHoldt, B. M. (2012). STRUCTURE HARVESTER: a website and program for visualizing STRUCTURE output and implementing the Evanno method. *Conservation Genetics Resources*, *4*, 359–361. https://doi.org/10.1007/s12686-011-9548-7

Evanno, G., Regnaut, S., & Goudet, J. (2005). Detecting the number of clusters of individuals using the software structure: A simulation study. *Molecular Ecology*, *14*, 2611–2620. https://doi.org/10.1111/j.1365-294X.2005.02553.x

Pritchard, J. K., Stephens, M., & Donnelly, P. (2000). Inference of population structure using multilocus genotype data. *Genetics*, *155*, 945–959. https://doi.org/10.1093/genetics/155.2.945
